# Supplementary material for: Resolving the full spectrum of human genome variation using Linked-Reads
Source: Genome Res. 2019 Apr;29(4):635–45. doi: 10.1101/gr.234443.118 (PMC6442396; doi:10.1101/gr.234443.118)
Supplement: Supplemental Material [file supp_gr.234443.118_Supplemental_longranger-2.2.zip › longranger-2.2.tar.bz2!/lr2.2-public/lib/stan/cmdstan/stan/doc/api/html/do__print_8hpp_source.html]

Stan: src/stan/common/do\_print.hpp Source File


|  |  |
| --- | --- |
|  | Stan  2.5.0  probability, sampling & optimization |


- Main Page
- Related Pages
- Namespaces
- Classes
- Files

- File List
- File Members

All Classes Namespaces Files Functions Variables Typedefs Enumerations Enumerator Friends Macros Pages

- src
- stan
- common

do\_print.hpp

Go to the documentation of this file.

1 #ifndef STAN\_\_COMMON\_\_DO\_PRINT\_HPP

2 #define STAN\_\_COMMON\_\_DO\_PRINT\_HPP

3

4 namespace stan {

5

6  namespace common {

7

25  bool do\_print(const int n, const bool special, const int refresh) {

26  return (refresh > 0) &&

27  (special || n == 0 || ((n + 1) % refresh == 0) );

28  }

29

43  bool do\_print(const int n, const int refresh) {

44  return do\_print(n, false, refresh);

45  }

46

47  }

48 }

49

50 #endif

stan::common::do\_print

bool do\_print(const int n, const bool special, const int refresh)

Indicates whether it should print on the current iteration.

**Definition:** do\_print.hpp:25


---

[ Stan Home Page ]

*© 2011–2014,
Stan Development Team.*
